# Cisplatin and vinorelbine first-line chemotherapy in non-resectable malignant pleural mesothelioma

**DOI:** 10.1038/sj.bjc.6604421

**Published:** 2008-06-10

**Authors:** J B Sørensen, H Frank, T Palshof

**Affiliations:** 1 Department Oncology, Finsen Centre/National University Hospital, 9 Blegdamsvej, Copenhagen DK-2100, Denmark; 2 Department Oncology, Aalborg University Hospital, Aalborg, Denmark; 3 Department Oncology, Aarhus University Hospital, Aarhus, Denmark

**Keywords:** malignant pleural mesothelioma, chemotherapy, vinorelbine

## Abstract

The aim was to evaluate the activity of cisplatin and vinorelbine in previously untreated, inoperable patients having histologically verified malignant pleural mesothelioma (MPM), normal organ function, and performance status 0–2. Treatment was vinorelbine 25 mg m^−2^ i.v. weekly and cisplatin 100 mg m^−2^ i.v. every 4 weeks with hydration and standard prophylactic antiemetic treatment. Patients gave written informed consent. Characteristics of 54 consecutive patients were: males 85%, epithelial subtype 74%, IMIG stages III and IV 35 and 46%, performance status 0, 1, and 2, 26, 69, and 6%, and median age 63 years (31–78 years). CTC grade 3 or 4 toxicity occurred with respect to leukocytopenia (48% of patients, grade 4 in 13%), nausea (13%), neurotoxicity (11%), nephrotoxicity (4%), and other toxicities (9%). There were no toxic deaths. The median number of cycles was four. The fraction of patients alive at 1-, 2-, and 3-years were 61, 31, and 4%, respectively, and median survival and median time to progression were 16.8 months (0.5 to 46.4 +months) and 7.2 months (1.6 to 40.6 + months). There were two CRs and 14 PRs (response rate 29.6%). Cisplatin and intravenous vinorelbine is a highly active regimen in MPM with a response rate and survival comparable to the most active regimens so far reported.

Malignant pleural mesothelioma (MPM) is a highly aggressive malignancy whose incidence is increasing throughout the world. The diffuse spreading growth of this neoplasm makes surgery difficult and hence an option for only a minority of the patients (Treasure and Sedrakyan, 2004). The treatment options for the majority of patients are best supportive care and palliative chemotherapy (Ceresoli *et al*, 2006a, 2006b). Chemotherapy for MPM is challenging, although several cytotoxic agents have been tested and the rates of objective tumour response have ranged from 10 to 30% with monotherapy (Tomek and Manegold, 2004). Cisplatin and carboplatin are both active and thus usually included in the most used combination chemotherapy regimens for MPM (Sørensen, 2008).

Pemetrexed is a multitargeted antifolate with a 14% response rate as a single agent in chemotherapy-naive MPM patients (Scagliotti *et al*, 2003). A recent randomised trial showed that combination chemotherapy with pemetrexed and cisplatin was superior to cisplatin single-agent treatment with respect to both time to progression and overall survival (Vogelzang *et al*, 2003). Raltitrexed is another antifolate tested in combination with cisplatin against cisplatin alone in a randomised trial (van Meerbeeck *et al*, 2005). The combination was superior compared to cisplatin alone with respect to time to progression but not with respect to overall survival. A recent randomised study compared active symptom control (ASC) alone to ASC plus vinorelbine and to ASC plus mitomycin plus vinblastine plus Cisplatin (Muers *et al*, 2007). The impact of chemotherapy on symptom control and quality of life have been evaluated in both of these randomised trials, pointing towards an improvement with respect to dyspnoea and stabilisation with respect to other parameters (Boyer *et al*, 2003; Bottomley *et al*, 2006). The activity of the chemotherapy is still modest, and hence novel agents need to be evaluated for use in combination chemotherapy regimens for improvement of outcome (Green *et al*, 2007).

A large number of single agents have been tested for activity in MPM (Berghmans *et al*, 2002; Ellis *et al*, 2006; Sørensen, 2008). The third generation vinca alkaloid vinorelbine has attracted interest in a phase II trial using vinorelbine 30 mg m^−2^ i.v. weekly (Steele *et al*, 2000). A cycle consisted of 6 weekly injections and the median number of injections was 12. The intention-to-treat response rate among 29 chemotherapy-naive MPM patients was 24% (95% confidence level 10–44%) and the fraction of patients alive 1 year from time of first treatment was 41%, which ranks vinorelbine among the most active agents in MPM. Toxicity was modest. On the basis of these results, it seems interesting and feasible to evaluate the activity of vinorelbine together with cisplatin. The feasibility and tolerability of this doublet is already very well known from its use in non-small cell lung cancer (NSCLC) in which it is among the most active regimens. Both the South West Oncology Group (SWOG) and the French group have published their data on activity and toxicity in NSCLC using cisplatin 100 mg m^−2^ every 4 weeks together with weekly i.v. vinorelbine 25 mg m^−2^ (Wozniak *et al*, 1998) or cisplatin 120 mg m^−2^ every 4–6 weeks together with weekly i.v. vinorelbine 30 mg m^−2^ (LeChevalier *et al*, 1994). Accordingly, the purpose of this study was to evaluate this regimen cisplatin and vinorelbine for its activity as first-line treatment in MPM using the regimen by SWOG.

## Materials and methods

### Patients

Eligibility criteria included histologically proven MPM, no previous chemotherapy, inoperable for anatomical or for physiological reasons, measurable disease, ECOG performance status 0–2, estimated survival expectancy of ⩾3 months, age ⩾18 years, and written informed consent.

Adequate organ functions were required, defined as WBC ⩾3000 *μ*l^−1^, platelets ⩾100 000 *μ*l^−1^, haemoglobin ⩾9.0 g per 100ml, bilirubin <1.25 times upper limit of normal, AST and ALT <2.5 times upper limit of normal, and creatinine <2.0 mg per 100ml. The renal function measured as a chrome-EDTA clearance had to be within normal limits.

Exclusion criteria included: Significant medical or psychiatric co-morbidity, central nervous metastases, pregnant or lactating women, and history of previous cancers in the previous 5 years or breast cancer ever. All patients of fertile capacity were to use safe contraception. The standards of Helsinki Declaration were fulfilled.

### Treatment

Cisplatin 100 mg m^−2^ was administered as a 1-h i.v. infusion together with intravenous hydration 1 l of normal saline before and after infusion and with standard antiemetic treatment using metoclopramide, prednisolone, and ondansetron every 4 weeks. Vinorelbine 25 mg m^−2^ was administered i.v. weekly as a 10-min infusion without routine antiemetic treatment. Weekly complete blood cell counts and chemistry panel were performed, and treatment was delayed by 1 week in the event of bone marrow suppression (WBC <3000 *μ*l^−1^, neutrophile count <1500 *μ*l^−1^ or platelets <100 000 *μ*l^−1^). No cisplatin was administered in case of decline in renal clearance to either severely reduced or less than 50 ml min^−1^. The patients did not receive vitamin substitution.

Dose was adjusted for grade 4 haematological toxicity, or grade 3 or 4 non-haematological effects were done. Dose delays up to 3 weeks were permitted for recovery from study drug toxicity. Dose escalations were not allowed. Granulocyte colony-stimulating factors were not routinely used.

### Assessments during treatment

Baseline and predosing assessments included complete history and physical examination, complete blood cell count, liver enzymes, blood electrolytes, blood albumin, calcium, and glucose. Measuring of renal function as chrome-EDTA clearance was performed at baseline and then before every second treatment cycle (every 8 weeks).

Spiral CT-scan was performed at baseline, before start of every other treatment cycle (every 8 weeks), and every 2 months after completion of study therapy. Chest X-ray was performed at baseline and before each treatment cycle. The staging system as defined by the International Mesothelioma Interest Group (IMIG) was used (Rusch, 1995). The new modified RECIST criteria for the assessment of response in MPM were applied (Byrne and Nowak, 2004). Change in disease was assessed by measuring the tumour thickness perpendicular to the chest wall or mediastinum in up to three involved areas of pleural rind at least 2 cm apart on computed tomography scan, at baseline, and at every other cycle (at least one measurement was >1.5 cm). A reduction of at least 30% on two occasions 4 weeks apart defined a partial response; an increase of 20% over the nadir measurement, progressive disease. A complete response (CR) was defined as the complete absence of all signs of disease without any new lesions or disease-related symptoms.

Survival was defined as the time from onset of treatment to the time of death from any cause. Time to progressive disease was defined as the time from onset of treatment until documented progression or death from any cause. For patients without any progression at the time of analysis, the date of last follow-up was considered right-censored.

### Statistical considerations

The current regimen was the only standard treatment available for this patient group at the time because pemetrexed was unavailable. Hence, it was not a formal phase II trial, though the treatment was approved by the participating institutions' local ethical committees and was prospective with the aim of data collection on predefined case report forms. Statistical analysis was to be done according to the Gehan one sample, two stage model (Gehan, 1961). Type I and type II errors were set at 5 and 20% and a minimum response rate of 20% would be of clinical interest in this population. Under this hypothesis, a total sample of at least 40 patients was required, but interim analysis at 14 evaluable patients would be performed to discontinue the study if 0 or 1 response was observed. To compensate for possible ineligibilities, some extra patients could be included. Kaplan–Meier curves were used to estimate the overall survival and time to progression.

## Results

### Patients' characteristics

Fifty-four consecutive patients were enrolled from February 2003 to September 2006. Most patients were males (85%), had epithelial subtype (74%), performance status 0–1 (94%), previous asbestos exposure (76%), and IMIG stages III–IV (81%) (Table 1). Median age was 63 years (range 31–78 years). Median lead time from initial diagnosis to start-of-study therapy was 52 days (range 1–118 days).

### Toxicity

A total of 204 treatment courses were administered, with a median of 4 (range 1–6). CTC grade 3 or 4 toxicity occurred with respect to leukopenia in 26 patients (48%), with 6 patients having grade 4 (11%) (Table 2). Five patients (9%) experienced febrile leukopenia and no septic deaths were encountered. None had grade 3 or 4 thrombocytopenia. Non-haematological grade 3 or 4 toxicity occurred with respect to nausea (13%), neurotoxicity (11%), nephrotoxicity (4%), and other toxicities such as tiredness or constipation in 9%. Nine patients (17%) were hospitalised owing to toxic effects of chemotherapy. One patient died within 30 days of treatment start owing to a pulmonary embolism.

Treatment intensity is shown at Table 3. The patients received in median 4 treatment courses and 80.4 and 77.2%, respectively, of planned cisplatin and vinorelbine doses. Dose reductions were necessary in 19 patients (35%), most frequently due to haematologic toxicity (15 patients) or nephrotoxicity (5 patients). Other less frequent causes were nausea, tinnitus, and hearing loss. These reasons were non-exclusive. Retreatment postponement due to delayed haematological recovery occurred in 21 patients (39%). Non-exclusive reasons were delayed haematologic recovery (10 patients), haematuria, pneumonia, and poor performance status.

### Response and survival

**Figure 1:**
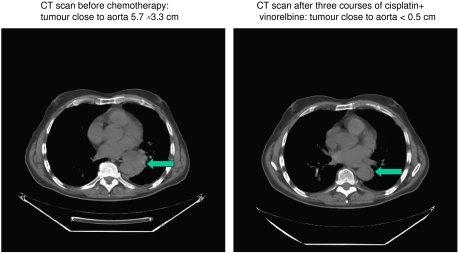
CT scan before and after three courses of cisplatin and vinorelbine in MPM with shrinkage of tumour close to aorta.

**Figure 2:**
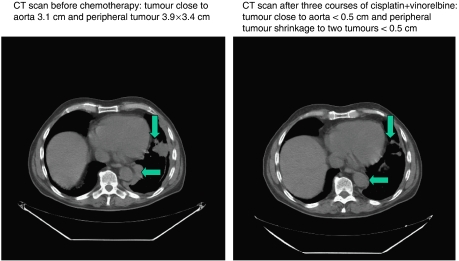
Same patients as Figure 1 with CT scan before and after three courses of cisplatin and vinorelbine in MPM with shrinkage of tumours more distally close to aorta and peripheral tumour.

Partial response and CRs occurred in 14 patients (25.9%) and 2 patients (3.7%), respectively, with an overall response rate of 29.6% (95% confidence limits 18.0–43.6%) (Table 4). A case of heavy tumour burden with regression after three treatment courses is shown in Figures 1 and 2. Thirteen responses occurred among the 40 cases having epithelial subtype (32.5%) and in two and one patients, respectively, among the five sarcomatous and nine biphasic cases. Four responses were noted among 8 female patients (50%) compared to 12 responses among 46 males (26%) (*P*=0.45).

The fractions of patients alive after 1 and 2 years were 61 and 31%, respectively (Table 4). Time to progression was in median 7.2 months, whereas median overall survival was 16.8 months.

**Figure 3:**
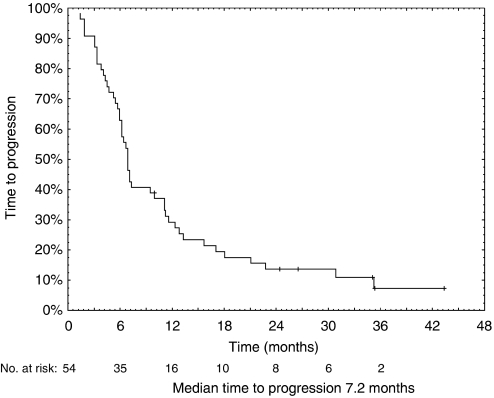
Kaplan–Meier curve of time to progression in 54 inoperable MPM patients treated with cisplatin and vinorelbine (median time to progression, 7.2 months).

**Figure 4:**
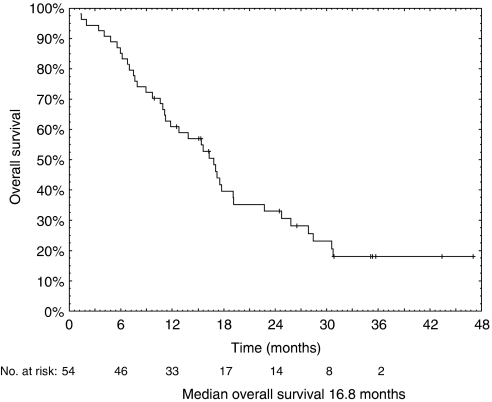
Kaplan–Meier curve of overall survival in 54 inoperable MPM patients treated with cisplatin and vinorelbine (median overall survival time, 16.8 months).

Curves of overall survival and time to progression are shown in Figures 3 and 4, respectively.

### Post-study treatment

A total of 22 patients (41%) received second-line chemotherapy (Pemetrexed 20 patients; carboplatin+caelyx+gemcitabine 2 patients). Seven patients received palliative irradiation.

## Discussion

This report provides data on the use of a combination of cisplatin and vinorelbine in chemotherapy-naive patients having inoperable MPM. The response rate of 29.6% is noteworthy, being comparable to the most used regimens in MPM (Table 4). It is, however, not possible to draw firm conclusions concerning major differences in activity between these regimens for several reasons. First, the majority are either phase II trials or retrospectively analyses except for the randomised trials by Vogelzang *et al*, 2003, and van Meerbeeck *et al*, 2005, which may not be entirely comparable as there is usually a tendency towards higher response rates observed in the very selective non-randomised trials. Another source of variability is the different mixture of various prognostic variables between the trials. Both analyses of prognostic variables by CALGB in 337 patients (Herndon *et al*, 1998), by EORTC in 204 patients (Curran *et al*, 1998), and in the randomised trial by van Meerbeeck *et al* in 250 patients pointed towards a better outcome for MPM patients having the epithelial histological subtype. A worse prognosis was reported for patients in poor performance status or advanced age. Independent predictors of survival in CALGB study were performance status, age, chest pain, weight loss, leukocyte count, and haemoglobin level, whereas they were histology, performance status, gender, and leukocyte count in the EORTC study. The frequency of epithelial subtype in the current study was 74%, which is somewhat higher than the around 60% usually considered average in the entire MPM population (Vogelzang, 2002), thus possibly contributing to a tendency towards a higher response rate. The frequency of epithelial subtype was, however, similar to the frequencies in newer studies, such as those by Vogelzang *et al*, 2003 (68% epithelial), van Meerbeeck *et al*, 2005 (75%), Berghmans *et al*, 2005 (74%), and van Haarst *et al*, 2002 (81%), pointing toward a rather uniform rate of epithelial subtype of 70–80% in more recent trials for chemotherapy in MPM and with the current study being in accordance with these trials. Interestingly, the activity is also comparable to that of single-agent Vinorelbine, which revealed a 24% partial response rate (Steele *et al*, 2000).

Measurement of tumour response to antineoplastic chemotherapy is notoriously difficult both when using the WHO and the RECIST criteria due to the parietal growth patterns of these tumours (Monetti *et al*, 2004; van Klaveren *et al*, 2004). Recently, modified RECIST criteria for use in MPM have been suggested by Byrne and Nowak, 2004, measuring tumour thickness perpendicular to the chest wall or mediastinum in two positions at three separate levels on thoracic CT scans. The use of these different systems in various reports may contribute to variations in the response rates observed, in addition to the variations caused by different distributions of known and unknown prognostic variables and possible differences in activity between the treatment regimens under evaluation. There is a growing evidence that therapy-induced changes in tumour FDG uptake, as measured by FDG-PET imaging, might predict response and patient outcome (Ceresoli *et al*, 2007). Thus, FDG-PET imaging could be potentially useful in the early assessment of treatment efficacy.

Haematological toxicity was relatively pronounced in the current study of cisplatin and vinorelbine with 48% of patients having leukopenia CTC grade 3 or 4 (Table 2). This is, however, in accordance with the 54% grade 3 or 4 leukopenia confined with cisplatin and epirubicin in MPM (Berghmans *et al*, 2005) and lower than the 81% of NSCLC patients who had grade 3 or 4 granulocytopenia in the randomised SWOG trial using same doses of cisplatin and vinorelbine as the current study (Wozniak *et al*, 1998). It is, however, considerably higher than was observed in the randomised trials in MPM patients with cisplatin and pemetrexed (18%, Vogelzang *et al*, 2003) or cisplatin and raltitrexed (7%, van Meerbeeck *et al*, 2005). Also, the rate of febrile leukopenia was somewhat higher, being 9% compared to 2 and 1%, respectively, in the two randomised trials cited above. There were no septic deaths and no toxic deaths overall, but the febrile lekopenia rate of 9% in this study is not only a potential risk but also an inconvenience for those patients who have to be admitted for intravenous antibiotics, as well as a cost for the health-care system. The use of granulocyte colony-stimulating factor may diminish the problem but was not a part of this protocol. Other toxicities were generally not pronounced (Table 2).

The survival of patients with MPM who received cisplatin and vinorelbine in this study was impressive with 31% being alive after 2 years and a median survival of 16.8 months, even though patients with adverse prognostic variables such as performance status 2 and age above 70 years were included (Table 1). The use of second-line chemotherapy in 22 patients may possibly have had an impact on survival. However, these figures are small and there are potential selection biases in the choice of second-line treatment or not. Hence, a possible impact cannot be explored or concluded from these data. The survival results compares favourably with those reported on for other active regimens, ranking the combination of cisplatin and vinorelbine among the most active cytotoxic treatments for MPM reported to date (Table 5). On the other hand, this regimen is convenient for neither the patients nor for the health-care system because of the weekly administration of intravenous vinorelbine and because of the haematological toxicity encountered. Other regimens in Table 4 may be more convenient and less troublesome in the palliative treatment setting. A number of regimens seem to possess similar activity without any regimen being clearly superior. No combination chemotherapy regimens have been compared to each other in randomised trials, but the regimens of both cisplatin and pemetrexed as well as cisplatin and raltitrexed have proved superior to single agent treatment with cisplatin in randomised trials (Vogelzang *et al*, 2003; van Meerbeeck *et al*, 2005). It is of great importance to use a treatment regimen with a high antitumour activity in another clinical situation such as induction chemotherapy before surgery, and the current regimen may, despite its inconvenience, be among the options for that situation.

The high activity of cisplatin and vinorelbine deserves attention for use as induction treatment before surgery in resectable cases. Further development of platinum compounds, together with vinorelbine in the palliative setting, also seems justified both in the light of the documented activity of the two-drug combination used in the current study and also because the single agent activity of vinorelbine with a response rate of 24% (Steele *et al*, 2000) ranks this agent among the most active single drugs in MPM. It must, however, be kept in mind that these results are obtained from relatively small and non-randomised trials with wide confidence limits. Improvement of the current regimen is necessary if it is to be used in the palliative situation to make it more feasible and carboplatin may thus be used instead of cisplatin and vinorelbine applied in the oral formulation. Further improvement of the regimen in the palliative setting is under evaluation by the investigative group. The oral formulation of vinorelbine may render the regimen more convenient and feasible, and evaluation of new targeted agents is necessary to improve prognosis for this dismal disease. Taking the documented activity of vinorelbine in the first-line treatment of MPM into consideration, vinorelbine may also be explored as second-line treatment for patients not previously exposed to this drug.

## Figures and Tables

**Table 1:** Characteristics of 54 MPM patients treated with cisplatin and vinorelbine

**Variables**	**No. of patients (%)**
Total	54 (100)
	
*Gender*	
Male	46 (85)
Female	8 (15)
	
*Asbestos exposure*	
No	11 (22)
Yes	41 (76)
NA	2 (4)
	
*Histology*	
Epithelial	40 (74)
Sarcomatous	5 (9)
Biphasic	9 (17)
	
*IMIG stage*	
Ia	1 (2)
Ib	1 (2)
II	8 (14)
III	19 (35)
IV	25 (46)
	
*Performance status*	
0	14 (26)
1	37 (69)
2	3 (6)
	
*Age*	
Median	63 years
Range	(31–78 years)

**Table 2:** Worst toxicity (CTC grading) in 54 MPM patients receiving cisplatin and vinorelbine

	**No. of patients (%)**				
**CTC grades**	**0**	**1**	**2**	**3**	**4**
*Variables*					
Leukocytes	6 (11)	4 (7)	18 (33)	19 (35)	6 (11)
Neutrophils	8 (15)	7 (13)	19 (35)	15 (28)	5 (9)
Thrombocytes	50 (93)	3 (5)	1 (2)	—	—
Nausea	19 (35)	21 (39)	7 (13)	7 (13)	—
Vomiting	31 (57)	12 (22)	11 (20)	—	—
Nephrotoxicity	26 (48)	12 (22)	14 (26)	2 (4)	—
Neurotoxicity	22 (41)	16 (30)	10 (19)	4 (7)	2 (4)
Other toxicity	4 (7)	24 (44)	21 (39)	4 (7)	1 (2)

**Table 3:** Intensity of treatment for 54 inoperable MPM patients treated with cisplatin and vinorelbine

**Variables**	
**No. of treatment courses**	**No. of patients (%)**
1	8 (15)
2	8 (15)
3	8 (15)
4	10 (19)
5	4 (7)
6	16 (30)
	
**Cumulative dose**	**mg m^−2^ week^−1^**
Cisplatin	20.1 (80.4%)^a^
Vinorelbine	19.3 (77.2%)^a^

a Percentage of cumulative dose relative to planned dose.

**Table 4:** Efficacy of cisplatin and vinorelbine in 54 inoperable MPM patients

**Variables**	**No. of patients (%)**
*Dead*	
No	12 (22)
Yes	42 (78)
	
*Fraction alive*	
1 year	33 (61)
2 years	17 (31)
	
*Survival*	
Median	16.8 months
(range)	(0.5 to 46.4+ months)
	
*Response*	
CR	2 (3.7)
PR	14 (25.9)
NC/PD	38 (70.4)
	
*Time to progression*	
Median	7.2 months
(range)	(1.6 to 40.6+ months)

**Table 5:** Selected combination chemotherapy regimens in MPM

**Author, year**	**Regimen**	***n***	**Response rate (%)**	**Median survival (months)**	**Time to PD (months)**	**1-year survival (%)**
Current study	CDDP+vinorelbine	54	29.6	16.8	7.2	61
van Meerbeeck, 2005 a	CDDP+raltitrexed	126	24.0	11.4	5.3	46
Vogelzang, 2003 a	CDDP+pemetrexed	226	41.3	12.1	5.7	51
Obasaju, 2007	CDDP+pemetrexed	728	20.5	10.8	NA	45
Santoro, 2007	CBDCA+pemetrexed	861	21.7	NA	NA	64
Ceresoli, 2006b	CBDCA+pemetrexed	102	18.6	12.7	6.5	52
Andreopoulou, 2004	CDDP+MMC+VBL	150	15.3	7.0	NA	31
Byrne, 1999	CDDP+gemcitabine	21	48.0	10.0	NA	NA
van Haarst, 2002	CDDP+gemcitabine	32	16.0	9.6	6.0	36
Favaretto, 2003	CBDCA+gemcitabine	50	26.0	14.7	8.9	53
Berghmans, 2005	CDDP+epirubicin	69	19.0	13.3	NA	50

Abbreviations: CDDP=cisplatin; CBDCA=carboplatin; MMC=mitomycin C; VBL=vinblastine; NA=not available.

a Data from randomised trial.
